# Filtering and Overlapping Data for Accuracy Enhancement of Doppler-Based Location Method

**DOI:** 10.3390/s25051465

**Published:** 2025-02-27

**Authors:** Rafał Szczepanik, Jan M. Kelner

**Affiliations:** Institute of Communications Systems, Faculty of Electronics, Military University of Technology, 00-908 Warsaw, Poland; jan.kelner@wat.edu.pl

**Keywords:** localization, Doppler effect, signal Doppler frequency (SDF), unmanned aerial vehicle (UAV), overlapping, digital filter

## Abstract

The localization of radio emitters is a fundamental task in reconnaissance systems, and it has become increasingly important with the evolution of mobile networks. The signal Doppler frequency (SDF) method, developed for dual-use applications, leverages Doppler frequency shifts (DFSs) in received signals to estimate the positions of radio transmitters. This paper proposes enhancements to the SDF method through advanced signal processing techniques, including dedicated filtering and a novel two-level overlapping approach, which significantly improve localization accuracy. The overlapping technique increases the number of DFS estimations per time unit by analyzing overlapping segments at both the signal sample level and within the DFS vector. Simulation studies using various filter types and overlapping parameters were conducted to evaluate the effectiveness of these enhancements in a dynamic scenario involving multiple stationary transmitters and a single moving receiver. The results demonstrate that the proposed approach minimizes localization errors. The application of low-pass filtering at the DFS vector level improves localization accuracy. In the study, three types of filters for different cutoff frequencies are considered. Each of the analyzed filters with an appropriately selected cutoff frequency provides a comparable reduction in localization error at the level of about 30%. The use of overlapping at the signal sample level with a factor of 10% allows for more than a twofold decrease in localization errors, while overlapping at the DFS vector provides an increase in the refresh rate of the position of localized objects. Comparative analysis with direct position determination techniques additionally showed high effectiveness of the SDF method, especially using data filtration and overlapping. The simulation studies carried out are of significant importance for the selection of the operating parameters of real localization sensors in unmanned aerial vehicle (UAV) equipment.

## 1. Introduction

The location of radio emitters is one of the basic tasks of reconnaissance systems [1,2]. The development of mobile networks contributed to the popularization of this process. The need for user positioning has resulted in location services (LCSs) becoming an element of second-generation (2G) and third-generation (3G) mobile networks. Primarily, LCSs were mainly used for optimal network operating or in emergency calls. LCSs have evolved towards location-based services (LBSs) in fourth-generation (4G) networks, i.e., Long-Term Evolution (LTE). In this case, estimating user position is widely utilized in various novel applications and services offered in mobile networks. Today, localization is an integral element/service of all modern radio communication networks [3,4].

The implementation of the location service in mobile networks requires providing an appropriate signal structure in which defined parameters are transmitted, e.g., round-trip time (RTT) [5,6,7]. Another approach is to measure such parameters independently of the transmitted signal structure. This approach is used in reconnaissance systems [1,2]. For the needs of these systems, the signal Doppler frequency (SDF) method has been developed [8,9], which can be implemented in dual-use applications. It uses changes in the Doppler frequency shift (DFS) in the received signal. Utilizing a mobile receiving platform, especially unmanned aerial vehicles (UAVs), provides this possibility [8,10].

During the localization process, the received signal must be adequately prepared to ensure appropriate accuracy of the signal parameter estimation constituting the basis of the selected localization technique. Hence, a common approach is using various digital signal processing methods. All radio communication systems use hardware (i.e., analog) [11] or software (i.e., digital) filtering [12] to minimize inter-channel interference [13]. In the SDF case, we recommend using additional filtering for the shape reconstruction of a Doppler curve (i.e., DFS versus time). Moreover, in this paper, we use an overlapping technique [14] that allows us to increase the number of estimated DFS values per time unit. The application of the proposed technique significantly improves the estimation accuracy and refresh rate of the radio emitter position based on SDF.

The SDF method has been used previously in a simplified form. Additional signal processing techniques enable higher localization accuracy. For the first time, we propose the application of dedicated filtering at the level of estimated DFS values. This paper compares three different types of filters. Moreover, we demonstrate the comprehensive impact of applying overlapping at two data processing levels: the signal samples used for DFS estimation and the DFS vector used for the estimation of the emitter position coordinates.

The remainder of the paper is organized as follows. Section 2 describes the basis of the SDF and a review of previous research. The ideas of DFS filtration and two-level overlapping in the SDF method are described in Section 3 and Section 4, respectively. The proposed solutions are evaluated based on the measurement scenario outlined in Section 5. The paper concludes with a summary of the findings.

## 2. Doppler-Based Location Method

In the literature, various classifications of localization methods are presented. The parameter of the received signal, which is the basis of the localization technique, is one of the most commonly used classification criteria [5,7,9]. In this case, we can distinguish time (e.g., time (TOA) or time difference of arrival (TDOA)), power (i.e., received signal strength (RSS)), angle (e.g., angle of arrival (AOA)), frequency (i.e., frequency (FOA), frequency difference of arrival (FDOA), or SDF), phase (i.e., phase of arrival (POA)), or hybrid methods, which are based on at least two different parameters. These are the so-called two-step methods. In the first step, signal parameter values are determined, and then the position of the located object is estimated. There are also one-step methods, known as direct position determination (DPD) [15,16], that are currently developing dynamically. However, in practice, two-step methods are dominant.

The SDF method has been being developed at the Military University of Technology for about 20 years. The analytical solution of the wave equation for a moving emitter allowed us to obtain a relationship between the instantaneous DFS, $f_{D}\left(t\right)$, and the spatial relations between the receiver (Rx) and transmitter (Tx) [17]:

(1) $$f_{D}\left(t\right)=f_{Dmax}\frac{x_{0}-vt}{\sqrt{{\left(x_{0}-vt\right)}^{2}+{y_{0}}^{2}+{z_{0}}^{2}}},$$

where $\left(x_{0},y_{0},z_{0}\right)$ is the actual Tx position in the local coordinate system related to the Rx motion along the X axis, v is the receiver velocity, $f_{Dmax}=f_{0}v/c$ is the maximum DFS, $f_{0}$ is the carrier frequency of the transmitted signal, and c is the lightspeed.

Transforming Equation (1) allows us to determine the Tx position relative to the local coordinate system. For a two-dimensional (2D) location, the estimated Tx coordinates, x and y, can be calculated from the following formulas [9]:

(2) $$x≅v\frac{t_{1}A\left(t_{1}\right)-t_{2}A\left(t_{2}\right)}{A\left(t_{1}\right)-A\left(t_{2}\right)},$$

(3) $$y≅\pm \sqrt{\frac{{\left[v\left(t_{1}-t_{2}\right)A\left(t_{1}\right)A\left(t_{2}\right)\right]}^{2}}{{\left[A\left(t_{1}\right)-A\left(t_{2}\right)\right]}^{2}}-z^{2}},$$

where

(4) $$\begin{array}{cc} A\left(t\right)=\frac{\sqrt{1-F^{2}\left(t\right)}}{F\left(t\right)}, & F\left(t\right)=\frac{f_{D}(t)}{f_{Dmax}} \end{array},$$

where $F\left(t\right)$ is the normalized DFS.

The analysis of Equations (2)–(4) shows that determining the coordinates $\left(x,y\right)$ requires DFS measurement at least of two intervals, $t_{1}$ and $t_{2}$. Furthermore, it should be assumed that the z coordinate is known. This simplified approach can be used, e.g., in the case of implementing an Rx on a UAV, which flies at a constant altitude (i.e., $z=z_{0}$). Three-dimensional (3D) localization using the SDF method is discussed in [8].

In the SDF method, a single Rx is enough to localize the radio emitter, while classical methods such as TDOA, TOA, or AOA are usually based on at least two or three sensors for 2D or 3D localization, respectively. Utilizing a moving measuring Rx, e.g., on a UAV, can introduce some difficulties in implementing the localization process. These are related to the need to accurately assign the current position with a specified time interval and the measured parameter. On the other hand, it also has some advantages. The estimation of the emitter position is performed by the Rx without the need for information exchange and data fusion from other sensors, which usually requires synchronization.

The first empirical studies with the SDF method were carried out using wheeled vehicles [18,19]. However, in the case of cars, their motion is limited by the street configuration and road regulations. Meanwhile, at a certain flight altitude, UAVs can generally move in any direction. This allows the appropriate selection of the Rx movement trajectory, on which the process of recording the signal emitted by the localized Tx is carried out. This can contribute to shortening the measurement time and increasing the accuracy of SDF localization. The potential of using UAVs in the SDF method was noticed early on. The first works focused on simulation studies, e.g., [8,20]. The development of the autonomous system of locating radio emitters (ASLER) [10] was an impulse for further empirical studies using UAVs.

Currently, we are implementing a project on “Command and control of a group of COMINT radio-electronic reconnaissance unmanned aerial vehicles based on modern IT technologies”, with the acronym UAV-COMINT [21], where COMINT means communication intelligence. As part of this project, we are developing and implementing a UAV technology demonstrator for SDF localization at the sixth technology readiness level (TRL). This solution uses the experience gained in the development and operation of ASLER.

In the case of Doppler effect-based localization methods, we have diagnosed three significant practical issues. One is the frequency stability of the radio sensor responsible for recording the received signal and determining the DFSs. In this case, in order to ensure the appropriate frequency stability, external-source clocks, such as rubidium or cesium frequency standards, are recommended. This topic is discussed in [21].

The second problem concerns determining the carrier frequency of the transmitted signal. According to Equations (2)–(4), information about $f_{0}$ is required to calculate the position of the located emitter. It may seem difficult to implement. In practice, we assume that this parameter is always estimated based on the analysis of the received signal. In particular, $f_{0}$ can be estimated by averaging the carrier frequency of the received signal. This is relatively simple in the case when the Tx is stationary and the Rx (i.e., the localization sensor) is placed on a mobile platform that can stop in place or hover in the air, e.g., a car or a copter-type UAV. Then, the Doppler effect does not occur, and $f_{0}$ can be estimated based on the spectra analysis of the received signal. The effectiveness of the SDF method for this variant has been confirmed in practical measurements [10,18,19].

In the case when the localization sensor is placed on a platform that is always in motion, e.g., a ship or a wing-type UAV, the aforementioned approach is difficult to apply. In such a case, we propose to use a so-called reference signal acquisition sensor, or a reference sensor for short, on a copter-type UAV that hovers in the air. This may estimate the carrier frequency of the received signal not burdened by the Doppler effect. This approach is being developed in the UAV-COMINT project [21], within which this research is being carried out.

In the case of a moving transmitter, the problem of estimating the carrier frequency of the transmitted signal is more complex, and requires analysis of the signal over a longer time period on the receiving side. The authors are currently developing an algorithm that would determine this parameter independently of the movement of the Tx or Rx. This is possible by appropriate selection of the localization sensor trajectory, and will allow us to eliminate the need for a reference sensor.

The third practical issue in the Doppler-based location method is the efficient processing of the recorded signals, which will ensure the appropriate information refresh rate and the accuracy of the position estimation of the localized object. This topic is taken up in this paper. The analysis conducted in the paper considers two important aspects of the SDF method. The first is the filtration of estimated DFSs, which primarily ensures the omission of gross errors and the minimization of fluctuations in instantaneous DFS values. The second aspect concerns the use of the overlapping algorithm idea. We propose its use at two levels of data processing, i.e., determining the DFS based on the spectra of signal samples, and estimating the emitter position using the SDF based on the DFS vector. We addressed this topic, although in a much narrower scope, in [22]. In that case, the application of overlapping focused primarily on the DFS vector level. On the other hand, the analysis referred to the variability of the Doppler curve in the full range of DFS changes, which is a special case. The analysis presented in the remainder of the paper is based on simulation studies. The aim of these studies is to develop efficient signal and data processing algorithms that will be implemented in the radio sensors of the developed UAV-COMINT system.

## 3. Filtering in SDF Method

### 3.1. Digital Filters

Digital signal filtration is a commonly used technique in digital signal processing that allows for the separation of signals, elimination of noise or interference, anti-aliasing, improvement of signal quality, and extraction of essential information, signal features, or properties.

Digital filters can be divided into low-pass, high-pass, band-pass, and band-stop filters, depending on which frequencies are attenuated or passed. In terms of structure, there are finite impulse response (FIR) and infinite impulse response (IIR) filters, where FIR filters are characterized by stability and a linear phase, and IIR filters by computational efficiency [12,14].

The fundamental filter parameters include the bandwidth, which defines the range of passed frequencies, and the cutoff frequency, at which significant signal attenuation occurs. Additionally, the slope of the attenuation characteristic and the level of ripple in the passband and stopband are important factors. The filter order defines the number of coefficients (for FIR) or poles and zeros (for IIR), and affects the steepness of the transition between bands—the higher the order, the better the filter selectivity, but also the greater the computational complexity. Proper selection of filter parameters allows for effective signal processing in various applications, such as communication, audio processing, or medical analysis [12,14,23].

FIR filters have a finite impulse response, meaning their reaction to a single-impulse input completely decays after a finite number of samples. The impulse response of an FIR filter is directly equal to its coefficients, and its transfer function can be expressed as a weighted sum of delayed input samples [12,14,23]:

(5) $$H\left(z\right)=\sum_{k=0}^{K}b\left[k\right]z^{-k},$$

where $b\left[k\right]$ represents the filter coefficients and K is the filter order.

IIR filters have an impulse response that, theoretically, never fully decays, due to feedback in the filter structure. Their transfer function is represented as a rational function [12,14,23]:

(6) $$H\left(z\right)=\frac{\sum_{m=0}^{M}c[m]z^{-m}}{1+\sum_{n=1}^{N}a[n]z^{-n}},$$

where c[m] and a[n] are the numerator and denominator coefficients, which define so-called the zeros (i.e., corresponding to the input signal) and poles (i.e., determining the feedback contribution) of the IIR filter, respectively; and N and M are the denominator and numerator orders, respectively.

### 3.2. Filtration in SDF Method

In the SDF method, we use typical filtering as used in radio signal processing. The main goal of this is to reduce noise and increase the signal-to-noise ratio (SNR), as well as anti-aliasing. In the case of simultaneous localization of multiple sources, it is necessary to separate sub-bands corresponding to signals coming from individual Txs and occurring at different frequencies [9]. Formally, we use band-pass filters for this purpose. This approach allows for the individual analysis of each signal coming from a specific Tx.

In our approach, DFSs are estimated based on the spectral analysis of the received signal. The accuracy of this estimation is of significant importance for localization errors in the SDF method. Therefore, we propose to apply additional low-band filtration at the DFS vector level. This vector can be treated as a signal slowly varying in time, associated with the Doppler curve. DFS errors result from imprecision of the applied signal estimation and processing method, and frequency instability of the Tx and Rx clocks. The estimated DFSs oscillate around the actual value, which can be related to the theoretical one defined by Equation (1).

To better illustrate the importance of low-band filtering the DFS vector, we first explain the concept of the Doppler curve with respect to theoretical and estimated DFSs in Section 3.3. Next, Section 3.4 defines the root mean squared localization error and cumulative distribution functions (CDFs) determined for the theoretical and estimated DFS errors. Finally, Section 3.5 allows us to justify the purpose of low-band filtration of the DFS vector.

### 3.3. Doppler Curves

The Doppler effect is a wave phenomenon that occurs when a Tx, an Rx, or another scattering-wave object moves. From the SDF viewpoint, a moving Rx with a stationary Tx and no scattering objects is the most desirable case. For this reason, the application of UAVs in the localization process provides better conditions than the use of wheeled vehicles [18,19]. Flying the UAV to a certain altitude can provide line-of-sight (LOS) or obscured LOS (OLOS) conditions [20]. On the other hand, a sufficiently high UAV speed and frequent refreshing of information about the emitter’s position allow for the location of objects that move at some speed [8].

**Figure 1:**
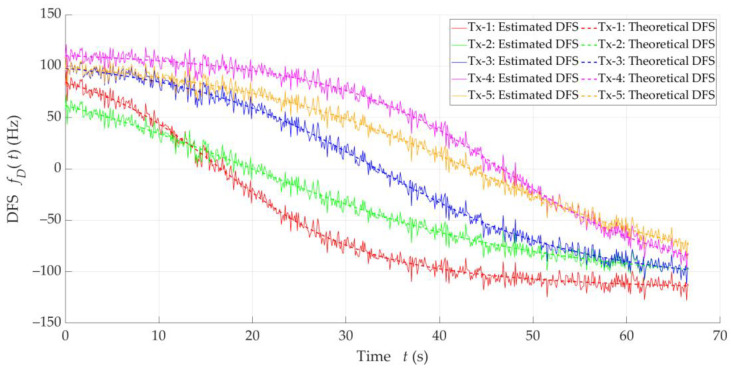
Exemplary Doppler curves (DFS versus time) for five emitters in different positions.

Based on Equation (1), we can conclude that the Doppler curve shape, i.e., the relationship between DFS and time, is a distinctive feature of the distance relationship between the Rx and Tx. Therefore, using the Doppler curve to locate emitters is very valuable. So, let us analyze exemplary Doppler curves for five hypothetical emitters located at different places, which are depicted in Figure 1. In fact, we have made the same assumptions as described in Section 5.

In Figure 1, we show the theoretical curves based on Equation (1) and sample estimated (i.e., empirical) curves, marked with dashed and solid lines, respectively. The latter were obtained from simulations, and consider DFS estimation errors resulting from propagation effects occurring in the radio channel between the Tx and Rx, frequency instabilities of radio devices, and signal processing. In this case, the frequency fluctuations are described by a zero-mean Gaussian distribution with a standard deviation (STD) of about 6 Hz. Similar DFS spreading values have been obtained in empirical measurements [10,21].

### 3.4. Location Error

To evaluate the localization accuracy, the error defined by the following formula is used [9]:

(7) $$\Delta r=\sqrt{{\left(x-x_{0}\right)}^{2}+{\left(y-y_{0}\right)}^{2}+{\left(z-z_{0}\right)}^{2}},$$

where $\left(x_{0},y_{0},z_{0}\right)$ and $\left(x,y,z\right)$ are the actual and estimated Tx coordinates, respectively.

The location error can be analyzed using the median (MED), the average value (AVG) and STD, the extreme values (i.e., minimum (MIN) and maximum), or the error distribution. In the following analysis, we use all of these, which allows us to evaluate the accuracy of the SDF method and proposed algorithms in a wider scope. Additionally, we show some examples of instantaneous errors.

**Figure 2:**
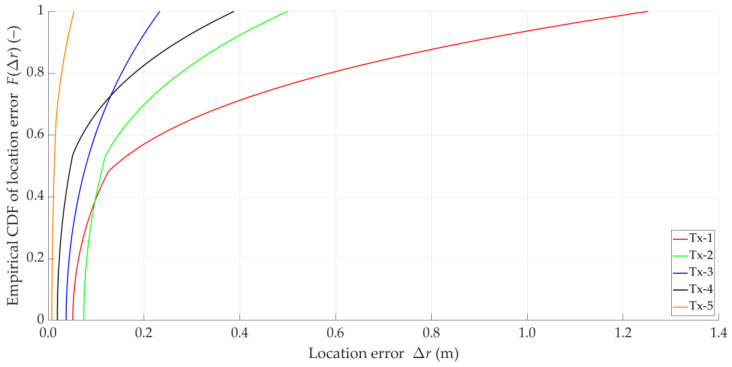
Empirical CDFs of location error for theoretical DFSs of five analyzed emitters.

Based on Equations (2)–(4) and (7), let us analyze the distributions of location error for the theoretical DFSs presented in Figure 1. Figure 2 depicts empirical CDFs of error, $F\left(\Delta r\right)$, defined by Equation (7), for the five analyzed theoretical Doppler curves.

The non-zero errors for the theoretical curves are due to the finite computational precision of the MATLAB R2024b environment in which the simulation was conducted. The visible diversification of error values is related to the different Tx locations. For this reason, in the rest of the paper, CDFs are determined as averaged for the five analyzed emitter locations. The above graphs illustrate the high effectiveness of the SDF method. On the other hand, they show a significant impact of the DFS estimation accuracy on location errors.

**Figure 3:**
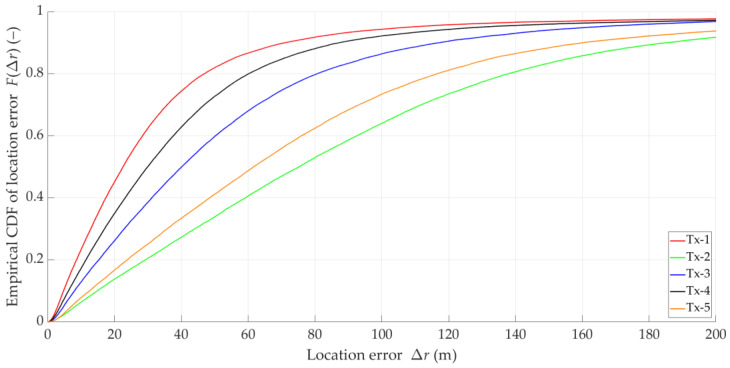
Empirical CDFs of location error for estimated DFSs of five analyzed emitters.

For comparison, we determined the empirical CDFs of the localization error for the five empirical Doppler curves (i.e., the estimated DFSs) from Figure 1. The CDF graphs are illustrated in Figure 3.

The above graphs show that the DFS estimation error significantly affects the accuracy of the SDF localization. This error is partially eliminated because the received signal is analyzed in a certain acquisition window (e.g., $T_{A}=5\;s$), which is explained in the description of the simulation studies (see Section 5). However, reducing the instantaneous DFS fluctuations can reduce the localization error. This fact is a justification for using DFS vector filtration. On the other hand, the graphs in Figure 2 and Figure 3 show that the character and range of the Doppler curve changes for a given Tx along the Rx motion trajectory affect the localization error in SDF. Therefore, in the remainder of the paper, we analyze the cumulative CDFs determined for the five analyzed Tx positions.

### 3.5. Idea of DFS Filtering

The analysis presented in Section 3.3 and Section 3.4 shows that the application of additional filtration in relation to the empirical DFS is justified. Based on the graphs in Figure 1, it may be concluded that the Doppler curves can be treated as signals slowly varying over time. Therefore, the applied filtration should be low-band. On the other hand, comparison of the corresponding CDFs from Figure 2 and Figure 3 shows the possibilities of improving the localization accuracy. Limiting the instantaneous fluctuations of the DFSs to bring them closer to the corresponding theoretical values can significantly reduce the localization error.

In the remainder of the paper, we analyze three types of filters, i.e., FIR filters with Hamming or Gaussian windows, and an IIR filter using the Butterworth approximation.

For a low-pass FIR filter, the ideal impulse response is defined by the sinc function [12,14,23]:

(8) $$h_{0}\left[k\right]=\frac{\sin\left(2\pi F_{C}\left(k-\left(K-1\right)/2\right)\right)}{\pi \left(k-\left(K-1\right)/2\right)},$$

where $F_{C}$ and K are the cutoff frequency and order of the filter.

The Hamming and Gaussian windows, respectively, are defined as described in [12,23]:

(9) $$w_{H}\left[k\right]≅0.54-0.46\cos\left(\frac{2\pi k}{K-1}\right),$$

(10) $$w_{G}\left[k\right]=\exp\left(-\frac{1}{2}{\left(\frac{k-\left(K-1\right)/2}{\sigma_{G}\left(K-1\right)/2}\right)}^{2}\right),$$

where $\sigma_{G}\leq 0.5$ controls the width of the Gaussian curve (typically $\sigma_{G}\approx 0.4$ to balance main lobe width and side lobe suppression), and k=0,1,2,…,K−1.

The final FIR filter impulse response is obtained by multiplying the ideal impulse response by the window [12,23]:

(11) $$h\left[k\right]=h_{0}\left[k\right]w\left[k\right],$$

where window $w\left[k\right]$ may be represented by $w_{H}\left[k\right]$ or $w_{G}\left[k\right]$.

The transfer function of the Nth-order Butterworth IIR filter is given by [12,23]:

(12) $$H\left(s\right)=\frac{1}{1+{\left(\frac{s}{\omega_{s}}\right)}^{2N}},$$

where s is the complex frequency variable in the Laplace domain, and $\omega_{s}$ is the cutoff frequency, where the gain drops to −3 dB.

We will show that an appropriate selection of the filter cutoff frequency improves the accuracy of DFS estimation and reduces the localization error. On the other hand, the results obtained for the analyzed filter types differ only slightly, which indicates that the filter type itself plays a negligible role.

## 4. Overlapping in SDF Method

### 4.1. Outline of Overlapping Technique Applications

The overlapping technique, also known as window overlap-add (OLA) or overlap-save, is a crucial technique in signal processing that enhances the accuracy of time–frequency analysis. This method involves dividing a signal into overlapping segments or windows, each of which is then individually analyzed. By ensuring that consecutive windows share common parts of the signal, the OLA method minimizes discontinuities and artifacts that can arise from windowing effects, thereby providing a more accurate representation of the signal characteristics over time [14].

In practice, the OLA technique is often employed in conjunction with the short-time Fourier transform (STFT), where a fixed-length window is slid over the signal with a specific overlap factor, typically 50%. This overlapping approach ensures that transient events and rapid changes within the signal are captured more effectively, enhancing the resolution of the time–frequency representation. The resulting segments are then processed, and the analysis results from each segment are combined, providing a comprehensive view of the signal behavior across time and frequency domains [14].

The OLA method has been widely applied in various fields. In speech processing, it is instrumental for accurate feature extraction, which is critical for speech recognition systems [24]. The OLA’s ability to capture fine temporal details helps to improve the recognition of phonemes and other speech components. Similarly, in audio signal processing [25], overlapping techniques are used for noise reduction [26], audio coding [27], digital audio effects [28], and music analysis [29]. For example, they enhance the performance of algorithms for beat detection and musical instrument identification by providing detailed temporal resolution [30]. In the literature, we can also find more complex OLA algorithms that are mainly used in audio and speech processing, e.g., the weighted OLA (WOLA) [31], synchronous OLA (SOLA) [28,32,33], pitch SOLA (PSOLA) [28,34,35], epoch-SOLA (ESOLA) [36], synchronized and adaptive OLA (SAOLA) [37], and peak alignment OLA (PAOLA) [38] algorithms.

Biomedical signal processing also benefits significantly from overlapping methods. It is used in the analysis of electroencephalogram (EEG) [39] and electrocardiogram (ECG) [40] signals to detect and diagnose various medical conditions. By applying overlapping windows, clinicians and researchers can achieve higher accuracy in identifying signal patterns and anomalies, leading to better diagnostic outcomes. Additionally, in wireless communications, overlapping techniques (especially WOLA) are employed in orthogonal frequency division multiplexing (OFDM) systems, which help to mitigate inter-symbol interference and improve data transmission reliability [41,42].

In radar signal processing, OLA algorithms, particularly the time-compression OLA (TC-OLA) approach, are employed to enhance the detection and resolution of targets by improving the accuracy of time–frequency representations of radar signals. This approach is particularly useful in scenarios where precise detection of moving targets or distinguishing between closely spaced objects is required. By applying overlapping windows, radar systems can better capture rapid changes and transient events within the received signals, thus improving range and Doppler resolution. The overlapping method is often used in conjunction with the STFT or other time–frequency analysis techniques in radar applications. By dividing the radar signal into overlapping segments, the method reduces the artifacts and inaccuracies that can arise from abrupt window transitions, leading to a more detailed and accurate representation of target characteristics such as velocity, range, and direction [43,44].

The classical OLA approach consists of averaging periodograms with the goal of noise variance reduction. Studies conducted by P. D. Welch [45] show that the use of the OLA algorithm with the overlapping factor equal to 50% (i.e., the overlap is half of the window length) reduces the noise variance by a factor of almost 2, due to the doubling of the number of sections. On the other hand, greater overlap (i.e., the overlapping factor is less than 50%) does not continue to reduce the variance, because the segments become decreasingly independent as the overlap increases [14]. The method of averaging the overlapping periodograms before estimating any signal’s properties is used in the abovementioned applications.

We propose to apply overlapping in a somewhat different way from the classical method. The two-level overlapping in the SDF method involves analysis at the levels of the received signal samples and estimated DFS vector. Primarily, this approach is used to multiply data and increase their refresh rate in dynamically changing conditions. On the other hand, it also enhances the accuracy of positioning emitters. In this case, the purpose of the applied overlapping at the sample level is not to reduce the influence of noise occurring in the radio channel, in contrast to the classical approach [14]. Similarly, noise variance reduction is not the primary goal of using the OLA method in audio effects [28]. For the SDF method, we assume that the SNR of the received signal provides the possibility of its detection and of DFS estimation. When there are dynamical DFS changes in the received signal, the application of the classical OLA idea at the sample level is not justified. In this case, it may cause the spreading of the received spectrum and increase the DFS estimation error. When the DFS changes are very slow (i.e., close to the maximum or minimum DFS) in the received signal, then the classical OLA method might be used.

In the proposed approach, the selection of window width (i.e., acquisition time) or overlap factor enables localization algorithm modifications to increase the refresh data rate and achieve more accurate results in estimating the emitter positions. In this case, using an overlapping factor of less than 50% may bring the expected results. The initial concept of the two-level overlapping algorithm in the SDF method was described in [22]. However, in that analysis, certain aspects were limited—no filtering was applied, only full Doppler curve variability was considered, and the overlap factor at the signal sample level was equal to 100%.

### 4.2. Idea of Two-Level Overlapping in SDF Method

**Figure 4:**
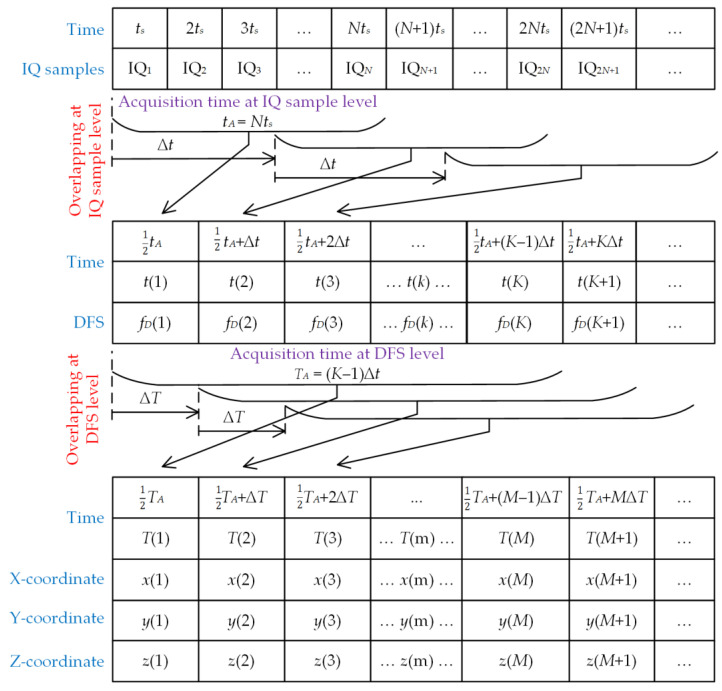
Double overlapping algorithm scheme.

Figure 4 and Algorithm 1 present the data processing scheme of the two-level overlapping dedicated to the SDF method.
**Algorithm 1** Two-level overlapping algorithm for SDF method**Require:** recording IQ (i.e., in-phase and quadrature components) samples of radio signal (S), sample rate ($F_{s}=1/t_{s}$), filter for DFS vector ($F_{A}$), measurement of instantaneous velocity and current position of Rx (e.g., based on GNSS).1. Set parameters at IQ sample processing level: initial indexes (k=1), initial time ($t\left(k\right)=0$), acquisition time at IQ sample level ($t_{A}=Nt_{s}$), number of IQ signal samples used for spectrum calculation (N), time step at IQ sample level ($∆t=t\left(k+1\right)-t\left(k\right)$), overlap factor at IQ sample level ($O_{t}=100\%\cdot ∆t/t_{A}$), signal spectrum resolution ($∆f=F_{s}/\left(P-1\right)$), number of spectrum samples (P).2. Prepare frequency (DFS) scale of analyzed spectrum calculated based on IQ signal samples: $F=\left[\begin{array}{cc} \begin{array}{cc} {-F}_{s}/2 & \left({-F}_{s}/2\right)+∆f \end{array} & \begin{array}{cc} \begin{array}{cc} \left({-F}_{s}/2\right)+2∆f & \cdots \end{array} & F_{s}/2 \end{array} \end{array}\right]$.3. Set parameters at DFS vector processing level: initial indexes (m=1), initial time ($T\left(m\right)=0$), acquisition time at DFS vector level ($T_{A}=\left(K-1\right)∆t$), number of DFSs used for Tx coordinate calculation (K), time step at DFS vector level ($∆T=T\left(m+1\right)-T\left(m\right)$), overlap factor at DFS vector level ($O_{T}=100\%\cdot ∆T/T_{A}$).  **repeat in parallel**   **repeat (every**
∆t**)**    4. Read S—IQ sample vector.    5. Extract subset of IQ samples: $S_{A}=S\left(t\left(k\right)-t_{A}:t\left(k\right)\right)$.    6. Compute amplitude spectrum based on Fourier transform: $W_{A}=\left|F\left(S_{A},P\right)\right|$.    7. From frequency scale, determine DFS value, where is maximum of signal spectrum: $f_{D}\left(k\right)=argument\left(max\left(W_{A}\right)\right)={\left.F\right|}_{max\left(W_{A}\right)}$.    8. Save $f_{D}\left(k\right)$to DFS vector $f_{D}$.    9. Increment index and time: k=k+1, $t\left(k\right)=t\left(k\right)+∆t$.   **until** $t\left(k\right)<t_{max}$.   **repeat (every**
∆T**)**    10. Read $f_{D}$—estimated DFS vector.    11. Extract subset of DFS vector: $f_{DA}=f_{D}\left(T\left(m\right)-T_{A}:T\left(m\right)\right)$.    12. Filtering subset of DFS vector: $f_{DF}=f_{DA}*F_{A}$.    13. Calculate Tx coordinates $\left(x\left(m\right),y\left(m\right)\right)$ based on Equations (2)–(4) for $f_{DF}$.    14. Increment index and time: m=m+1, $T\left(m\right)=T\left(m\right)+∆T$.   **until** $T\left(m\right)<T$.  **until stop**.

The mobile platform with the Rx provides ongoing recording of IQ signal samples. Currently, the Rx is usually made using software-defined radio (SDR) technology, while a UAV is used as a mobile platform. Additionally, the localization system should provide a measurement of the instantaneous velocity and position of the Rx. The global navigation satellite system (GNSS) receiver included in the mobile platform is generally used for this purpose [18].

The implementation of the two-level overlapping algorithm requires, first of all, defining the basic time parameters of data processing:

- The acquisition time $t_{A}$ and time step Δt at the IQ sample level;
- The acquisition time $T_{A}$ and time step ΔT at the DFS vector level.

#### 4.2.1. Overlapping at IQ Sample Level

The SDR platform provides the acquisition of IQ samples of the received signal, which are written to a vector S. From this vector, a sample subset, $S_{A}$, with length N and the acquisition time equal to $t_{A}$, is extracted. Then, for the vector $S_{A}$, the amplitude spectrum $W_{A}$ is calculated using the fast Fourier transform (FFT). The resulting spectrum has P frequency values ranging from ${-F}_{s}/2$ to $F_{s}/2$ at the resolution Δf. The instantaneous DFS, $f_{D}\left(k\right)$, is determined as the argument of the spectrum for which the maximum value occurs. The estimated DFSs are written to the vector $f_{D}$.

The above procedure is repeated every Δt, so the overlap factor at the IQ sample level is equal to

(13) $$O_{t}=100\%\cdot \Delta t/t_{A}$$

#### 4.2.2. Overlapping at DFS Vector Level

Determining the Tx position based on the SDF is carried out in parallel with the DFS estimation procedure based on the spectrum analysis. At this data processing level, the overlapping algorithm is also introduced. The initialization of the Tx coordinate determination process can occur at the moment of filling the vector $f_{D}$ by K values of DFS. A subset of the DFS vector, $f_{DA}$, with length K and the acquisition time $T_{A}$, is extracted from the vector $f_{D}$. Next, this subset is convolved with a low-pass filter $F_{A}$, which is designed to reduce the fluctuations of the instantaneous DFS. Based on the filtered vector $f_{DF}$, Tx coordinates, $\left(x\left(m\right),y\left(m\right)\right)$, are calculated using Equations (2)–(4).

The above procedure is repeated every ΔT, so the overlap factor at the DFS vector level is equal to

(14) $$O_{T}=100\%\cdot \Delta T/T_{A}$$

## 5. Simulation Studies

The simulation studies were generally realized for the scenario and assumptions presented in Section 5.1. The evaluation of the impact of the low-pass DFS filtration is contained in Section 5.2. Section 5.3 and Section 5.4 draw the impact evaluations of the time step at the IQ sample and DFS vector levels, respectively, whereas the influence of the acquisition time at the DFS vector level is analyzed in Section 5.5. Finally, in Section 5.6, we compare the performance of the proposed solution, i.e., the SDF, with different DPD methods, considering the DFS filtration and data overlapping. In this case, the simulation scenario proposed in [15] is used.

### 5.1. Spatial Scenario and Assumptions

**Figure 5:**
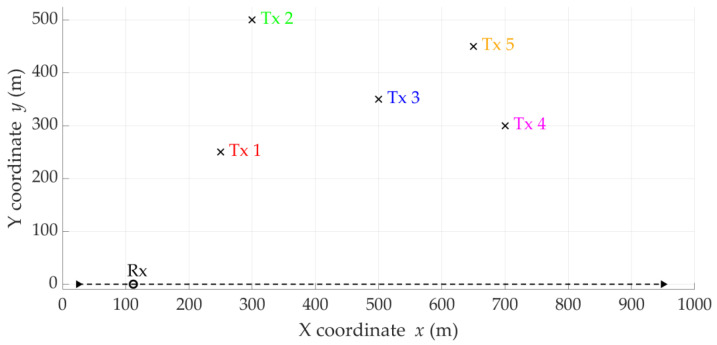
Simulation scenario with moving Rx and five stationary Txs.

A simulation study was conducted to evaluate the effectiveness of filtration and overlapping. To this aim, we analyzed the scenario with five emitters (Txs), which is depicted in Figure 5. These Txs are located on the flat surface of the earth in the following positions: $\left(x_{0},y_{0}\right)=\left[\left(250,250\right),\left(300,500\right),\;\left(500,350\right),\left(700,300\right),\left(650,450\right)\right]\left(m\right)$. The Rx placed on the UAV moves rectilinearly along the OX axis at a constant speed of v=15 m/s and at an altitude of ${h=z}_{0}=100\;m$. The length of the UAV flight trajectory is 1000 m, and starts at the origin of the coordinate system. We assumed that each Tx generates a monochromatic signal on different carrier frequencies in the ultra-high frequency (UHF) band: $f_{0}$=$\left[2335.1,\;2335.2,\;2335.3,\;2335.4,\;2335.5\right]\left(MHz\right)$. In this case, the signals generated by all Txs can be received by SDR platforms with a bandwidth of about 500 kHz.

The Doppler curves for the five analyzed Tx positions and the assumptions made are presented in Figure 1. In this case, the theoretical and estimated DFS curves are shown by dashed and solid lines, respectively. The theoretical ones are obtained based on Equation (1). To model the random fluctuations of the DFS around a mean value corresponding to the theoretical DFS, we used an uncorrelated Gaussian process with zero mean and some STD. This is a typical approach used in modeling the Doppler effect for signals received under LOS conditions [46,47]. In our studies, we determined the STD value (i.e., $\sigma_{f_{D}}=6\;Hz$) based on the measurement results obtained for a typical SDR, which we use in a UAV scenario [10,21]. This approach is commonly used in simulation studies regarding Doppler effect-based techniques. Then, the DFS estimation error considers both the inaccuracy of the spectra analysis process and the instability of the Tx and Rx clocks. On the other hand, the adopted assumptions correspond to the spectral analysis conditions described in [8], i.e., the sample rate is $F_{s}=1\;MS/s$, the bandwidths of the received signal and decimation filter are equal to 500 kHz and 10 kHz, respectively, the spectrum resolution (i.e., the basic frequency of signal analysis) is equal to 0.05 Hz, and the minimum SNR is equal to 3 dB.

The shape of the Doppler curve is related to the spatial relations between the Tx and Rx. For $f_{D}=0$, there is a so-called point of closest approach (PCA), at which point an Rx is located closest to a Tx. If the Tx–Rx distance at the PCA is smaller, then the Doppler curve slope is steeper. Therefore, at more considerable Tx–Rx distances at the PCA, the DFS curves have a gentler course. We may see this, e.g., by comparing the graphs in Figure 1 for Tx-1 and Tx-2.

### 5.2. Simulation Results for DFS Filtration

**Figure 6:**
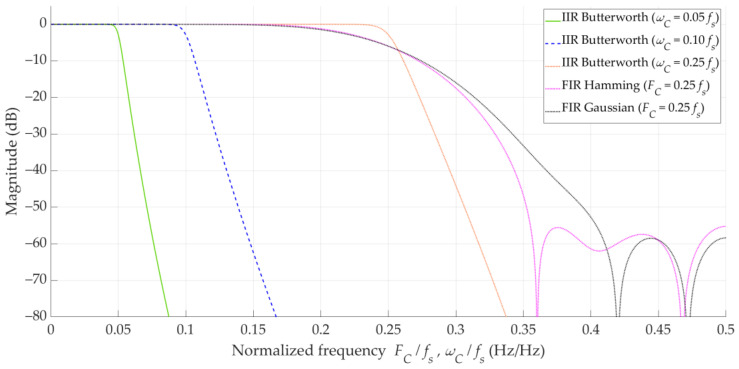
Estimated and filtered Doppler curves for five emitters in analyzed scenario.

Using the Filter Designer tool from the MATLAB R2024b. environment, we generated the three filters defined in Section 3.5, i.e., the two FIR filters with the Hamming and Gaussian windows, and the IIR filter using the Butterworth approximation. These filters are characterized by the same order, i.e., K=N=16, and the same cutoff frequency. In the comparative analysis, we considered three cutoff frequencies, i.e., $F_{C}=\omega_{C}=\left\{0.05,\;0.1,0.25\right\}\cdot F_{s}$, where $F_{s}$ is the sample rate. Figure 6 depicts the frequency responses of the designed filters. In this case, we illustrate the IIR filter for three different cutoff frequencies, while the FIR filters are illustrated only for $F_{C}=0.25F_{s}$.

Butterworth filters are characterized by a maximally flat amplitude response in the passband, but a relatively gradual transition between the passband and stopband in relation to other types of IIR filters (e.g., Chebyshev of elliptic). However, when comparing the slopes of these filters to that of the analyzed FIR filters, they are steeper [23].

The Hamming window (similar to Hann, Blackman, and Kaiser windows) is used to minimize the Gibbs phenomenon (which occurs due to abrupt truncation of the ideal response) and improve the transition between bands. It provides a moderate roll-off in the transition band and good (about −53 dB) attenuation in the stopband [23].

The Gaussian window provides optimal time–frequency localization, meaning it minimizes the product of temporal and spectral spread. It reduces spectral leakage and offers a smoother roll-off in the frequency domain than the Hamming window. Unlike the Hamming window, it has no side lobes if truncated correctly, making it useful in certain precision filtering applications [23].

We compared the efficiency of DFS filtration on two levels. In the first step, we analyze how filtration affects the reduction of the absolute DFS error,

(15) $${\Delta f}_{D}=\left|f_{D}-{\tilde{f}}_{D}\right|,$$

where $f_{D}$ is the theoretical DFS calculated based on Equation (1), and ${\tilde{f}}_{D}$ is the estimated (empirical) or filtered DFS. In the next step, we observed how this translates into the localization error, Δr. Table 1 compares the absolute DFS errors for the empirical data (i.e., without filtering) and the three analyzed filter types with three different cutoff frequencies. In this case, the MED, AVG, and STD of ${\Delta f}_{D}$ are used as comparative measures.

The presented results show that the use of filtration provides an improvement in the DFS estimation accuracy in relation to the theoretical value. The DFS error decreases with reduction in the cutoff frequency. In general, for the three analyzed filters with the selected cutoff frequencies, the error values are similar. This may be due to the fact that the Doppler curves are slowly changing signals, and the slope of the transition between bands or the attenuation level of the side lobes plays a relatively minor role. However, it should be noted that the Butterworth IIR filter provides the best filtration efficiency. See that a fivefold reduction in the cutoff frequency provides about a twofold reduction in ${\Delta f}_{D}$. This is probably due to it having the highest slope of transition between bands among the analyzed filters.

**Figure 7:**
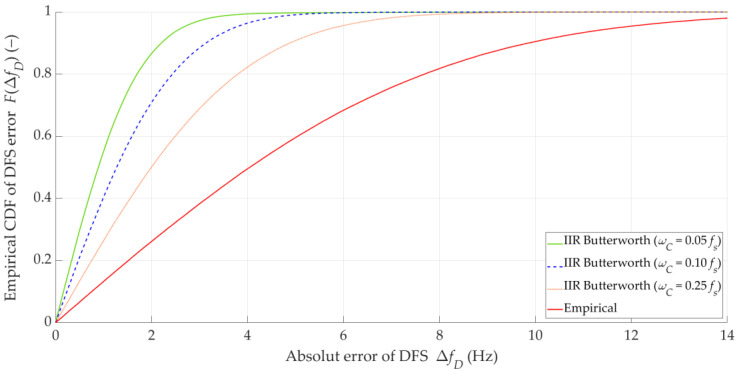
Empirical CDFs of absolute DFS error for empirical DFS vector and Butterworth IIR filters with three selected cutoff frequencies.

The DFS error distribution may provide a more complete analysis than individual parameters. Since the parameters obtained for the individual filter types are similar, in further analysis, we only show graphs for the IIR filters. Figure 7 shows the empirical CDFs of the absolute DFS error, $F\left({\Delta f}_{D}\right)$, for the empirical DFS vector, and the Butterworth IIR filters with three selected cutoff frequencies.

**Figure 8:**
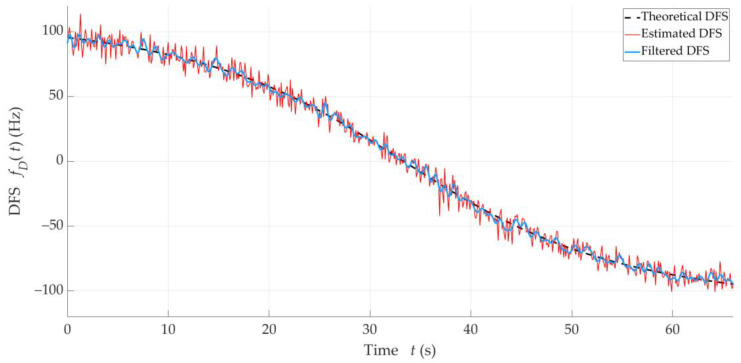
Theoretical, exemplary empirical, and filtered Doppler curves for Tx-3.

The result of the filtering application should also be presented directly on the Doppler curve. Figure 8 illustrates three Doppler curves, i.e., a theoretical one, an exemplary empirical one (i.e., DFS estimated from spectral analysis), and a curve at the output of the IIR filter with the cutoff frequency of $\omega_{C}=0.05F_{s}$. These curves correspond to Tx-3.

**Figure 9:**
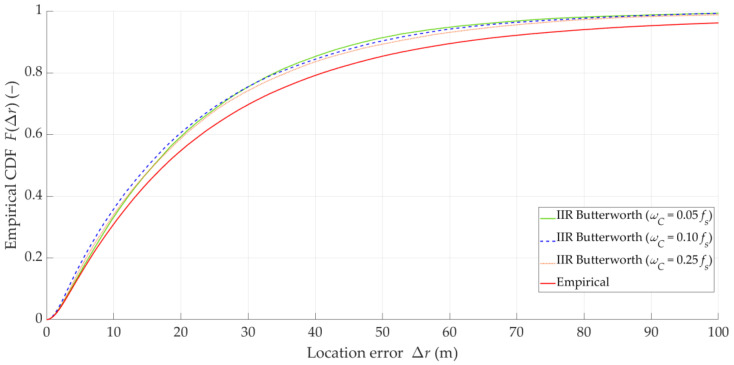
Empirical CDFs of location error for estimated (empirical) data and Butterworth IIR filters with three selected cutoff frequencies.

We also evaluated the impact of DFS filtering on the localization error. The results for the three considered filters and the unfiltered DFS vector (empirical) are presented in Table 2. In this case, the MIN, MED, AVG, and STD of Δr are determined for $F_{C}=\omega_{C}=0.05F_{s}$, $t_{A}=\Delta t=0.1\;s$, and $T_{A}=\Delta T=10\;s$ (i.e., no overlapping). Figure 9 depicts the empirical CDFs of the location error, $F\left(\Delta r\right)$ for the estimated (empirical) data and the Butterworth IIR filters with the three selected cutoff frequencies.

The results in Table 2 show that the utilization of different low-pass filters provides similar improvements in the SDF localization accuracy. This approach provides a reduction in the AVG error by about 30% and a reduction in the STD of Δr by a magnitude order, whereas the MED of Δr decreases only by about 2 m. Furthermore, Figure 9 depicts that the application of filters with different cutoff frequencies introduces small differences in the location error distributions. This may be due to the aforementioned fact that the Tx position is determined based on the DFS vector corresponding to the acquisition time, $T_{A}=10\;s$, over which the averaging takes place. Hence, the DFS fluctuations have some effect on the SDF accuracy, but this effect is more significant for short acquisition times. In further studies, we use the Butterworth IIR filter with $\omega_{C}=0.05F_{s}$.

### 5.3. Simulation Results for Overlapping at IQ Sample Level

Based on Algorithm 1 described in Section 4.2, the overlapping approach at the IQ sample level was tested for the adopted assumptions. The acquisition time at this level is $t_{A}=0.1\;s.$ We analyze four time steps at the IQ sample level, i.e., $\Delta t=\left\{0.01,\;0.02,\;0.05,\;0.1\right\}\left(s\right),$ so the adequate overlap factor may be equal to $O_{t}=\left\{10,\;20,\;50,\;100\right\}\left(\%\right)$. In order not to consider the overlapping at the DFS vector level, the following parameter values are adopted: the acquisition time and time step at this level are equal to $T_{A}=10\;s\;$and ΔT=10 s, respectively, whereas the overlap factor at the DFS vector level is $O_{T}=100\%$.

**Figure 10:**
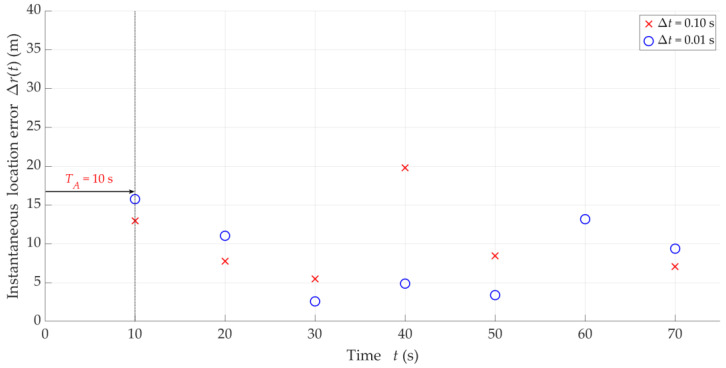
Examples of instantaneous location errors for Tx-3 and two selected Δt along Rx trajectory.

**Figure 11:**
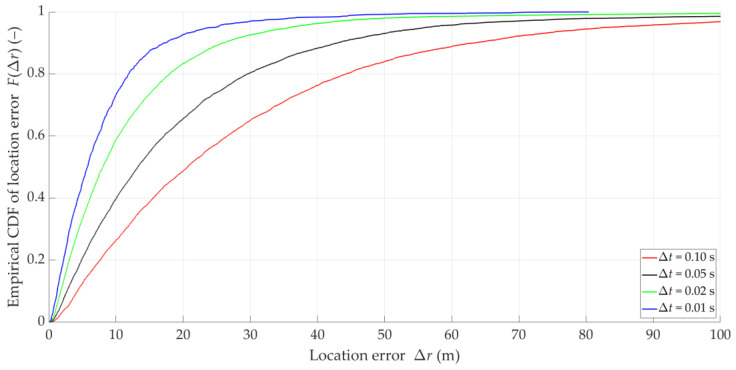
Empirical CDFs of location error for four selected time steps at IQ sample level.

Examples of instantaneous location errors for Tx-3 and two selected Δt, determined along the Rx trajectory, are illustrated in Figure 10. The MIN, MED, AVG, and STD of Δr for four analyzed Δt are included in Table 3. Figure 11 depicts the empirical CDFs of Δr, which allows for a wider assessment of the impact of overlapping at the IQ sample level (i.e., for different Δt) on the SDF accuracy. In this case, the parameters and distributions are averaged for the five analyzed Txs.

The obtained results show that decreasing $O_{t}$ (or Δt for constant $t_{A}$) generally reduces localization errors Δr, i.e., improves the SDF accuracy. Decreasing Δt tenfold reduces the average Tx positioning error by 2–3 times. On the other hand, reducing $O_{t}$ contributes to the densification of the determined DFSs. However, it is worth highlighting that improving the accuracy of the analyzed method cannot be achieved by permanently decreasing the overlap factor at the IQ sample level. The curves for $O_{t}$ equal to 10% and 20% are already close to each other.

Based on the obtained results, we recommend using the overlapping at the IQ sample level with $O_{t}=10\%$. When selecting the overlapping parameter values at this level, the limitations resulting from the capabilities of the equipment used on the UAV (e.g., a field-programmable gate array (FPGA) in SDR, or a microcomputer used for calculating the FFT) should always be considered. The computing power of UAV on-board devices does not currently allow for overlapping algorithms with arbitrary parameters.

### 5.4. Simulation Results for Overlapping at DFS Vector Level

In this study, we used Algorithm 1, the assumptions, and the Butterworth IIR filter described in Section 4.2, Section 5.1 and Section 5.2, respectively. Overlapping at the IQ sample level was disabled, i.e., $O_{t}=100\%$ and $t_{A}=\Delta t=0.1\;s$. The acquisition time at the level of processing the DFS vector is $T_{A}=10\;s$. We analyze four time steps at this level, i.e., $\Delta T=\left\{1,\;2,\;5,\;10\right\}\left(s\right),$ so the adequate overlap factor may be equal to $O_{T}=\left\{10,\;20,\;50,\;100\right\}\left(\%\right)$.

**Figure 12:**
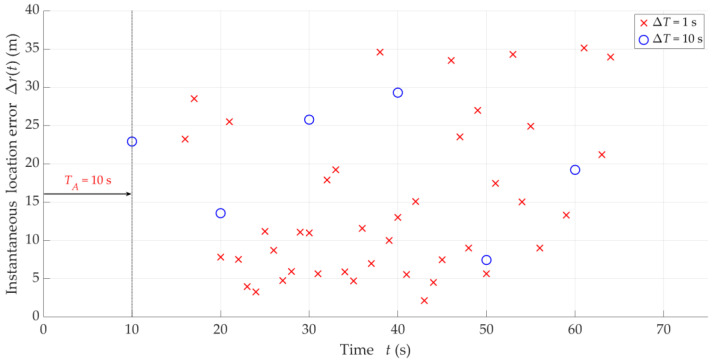
Examples of instantaneous location errors for Tx-3 and two selected ΔT along Rx trajectory.

**Figure 13:**
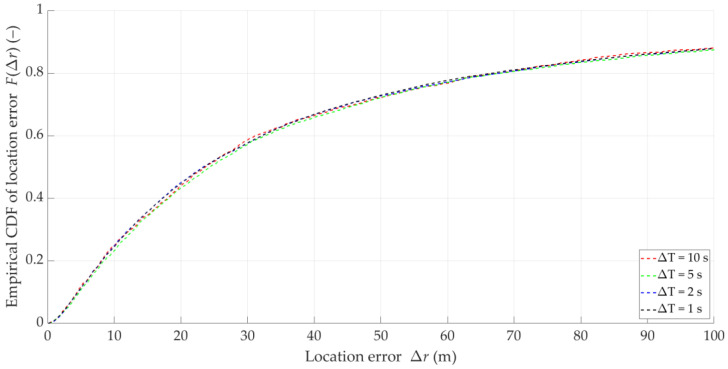
Empirical CDFs of location error for four selected time steps at DFS vector level.

In this scenario, we present the results in a similar way as in Section 5.3. Figure 12 presents samples of instantaneous location errors for Tx-3 and two selected ΔT determined along the Rx trajectory. Table 4 contains the MIN, MED, AVG, and STD of Δr for the four analyzed ΔT, whereas, the empirical CDFs of the location error, $F\left(\Delta r\right)$, are shown in Figure 13. This provides an evaluation of the influence of overlapping at the DFS vector level (i.e., for different ΔT) on the localization accuracy. In this case, the parameters and distributions are also averaged for the five analyzed Txs.

In the analyzed scenario, the obtained results show that the overlapping algorithm at the DFS vector level does not improve the SDF accuracy. The varying parameter ΔT, which increases the number of estimated coordinates $\left(x,y\right)$, does not lead to a reduction in the accumulated error Δr. This is related to the fact of averaging DFSs in a window of $T_{A}$ duration when determining the localized object position. At small $T_{A}$ values, when the number of the considered discrete DFSs is small, the overlapping algorithm at this level can be of significant importance.

So, what is the justification for using overlapping at this level of data processing? In this case, overlapping provides an increase in the refresh rate of determining the emitter position. This is crucial in real localization systems. If overlapping is not applied, then the position may not be sufficiently up-to-date for a large $T_{A}$. In the case of stationary Txs, this does not matter much, in contrast to the scenario with mobile emitters (see [8]). Thus, to sum up, overlapping at the IQ sample level improves localization accuracy, while overlapping at the DFS level provides an appropriate refresh rate of the localized object positions.

### 5.5. Impact of Acquisition Time at DFS Level on SDF Accuracy

To examine the impact of the acquisition time $T_{A}$ on location accuracy, we conducted an additional simulation study. In this study, we adopted the previous simulation assumptions: a Gaussian filter and overlapping at the IQ sample level of $O_{t}=10\%$. Furthermore, we assumed that ΔT=10 s and $T_{A}=\left\{5,\;10,\;20,\;30\right\}\left(s\right)$, so the overlapping factors at the DFS vector level are $O_{T}=\left\{200,\;100,\;50,\;33\right\}\left(\%\right)$, respectively.

**Figure 14:**
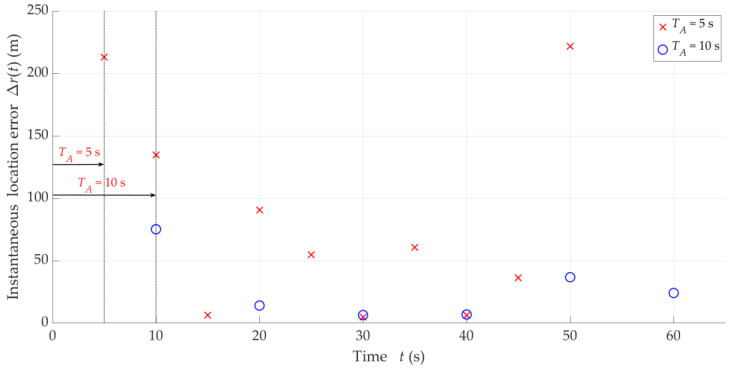
Examples of instantaneous location errors for Tx-3 and two selected $T_{A}$ along Rx trajectory.

**Figure 15:**
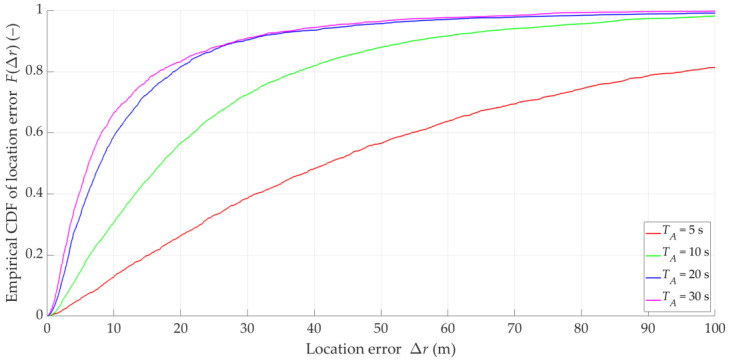
Empirical CDFs of location error for four selected acquisition times at DFS vector level.

The result analysis in this scenario is based on the scheme used in previous scenarios. In Figure 14, examples of instantaneous location errors for Tx-3 and two selected $T_{A}$ determined along the Rx trajectory are depicted. The MIN, MED, AVG, and STD of Δr for four selected $T_{A}$ are presented in Table 5. Figure 15 draws the empirical CDFs of the location error, $F\left(\Delta r\right)$, accumulated for the five Txs. In this case, we can observe the result of applying the proposed filtration and two-level overlapping for the different $T_{A}$.

The results indicate that increasing the acquisition time generally contributes to increasing the positioning accuracy. This is due to the fact that increasing the $T_{A}$ increases the DFS variability range, which translates into a reduction in localization errors in the SDF. In theory, the best results can be obtained for the full Doppler curve variability (see [22]). However, considering the practical aspects of measurements, a long acquisition time can contribute to the appearance of additional errors resulting from the frequency instability of the Tx or Rx (see [21]), the movement of localized objects (see [8]), or changes in the Rx flight parameters (e.g., speed, trajectory).

### 5.6. Comparison of Developed Approach with DPD Techniques

**Figure 16:**
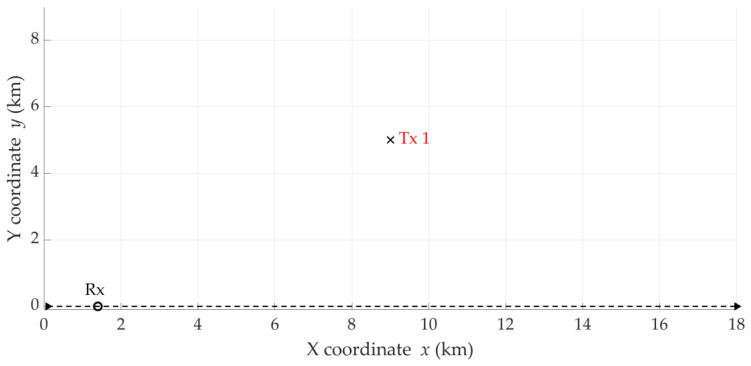
Simulation scenario with moving Rx and single stationary Tx.

To compare the performance of the proposed solution, i.e., SDF considering DFS filtration and data overlapping, with different DPD methods, the 2D simulation scenario proposed in [15] is adopted. In this case, a single emitter (i.e., Tx) is located on the flat earth surface at the following position, $\left(x_{0},y_{0}\right)=\left(9,\;5\right)\left(km\right)$. The Rx mounted at the UAV moves along the OX axis at a constant speed of v=300 m/s. The length of the UAV flight trajectory is 18 km, and it starts at the origin of the coordinate system. Figure 16 illustrates this situation. In the source scenario [15], two mobile sensors (i.e., Rx) are used to localize the Tx on the plane. In the analyzed case, we use only the single mobile Rx, which is a characteristic feature of the SDF method. From a formal viewpoint, the second stationary Rx could act as a reference sensor (see Section 2). The carrier frequency of the transmitted signal is $f_{0}=0.1\;GHz$, and the SNR is equal to 0 dB. The results are obtained based on 5000 Monte Carlo trials. The remaining assumptions are as in Section 5.1.

In the aforementioned research [15], four DPD methods were used, i.e., expectation maximization DPD (EM-DPD) [48], modified EM DPD (MEM-DPD) [15], maximum likelihood DPD (ML-DPD) [49], and quasi-Newton DPD (QN-DPD) [50].

The EM algorithm is an iterative method for finding maximum likelihood estimates of parameters in statistical models with incomplete or missing data. It consists of two steps: the expectation step (E-step), which calculates the expected value of the log-likelihood, and the maximization step (M-step), which updates the parameters to maximize this value. The process repeats until convergence. It is widely used in signal processing, pattern recognition, and statistical analysis due to its versatility, though it may converge slowly in some cases [48].

The MPM algorithm is an enhanced version of the EM method, specifically designed for DPD. The MEM improves both the accuracy and convergence speed compared to the standard EM by refining the iterative process. It is particularly effective in navigation and localization applications, offering robust performance in scenarios with multiple signal sources [15].

The ML method estimates model parameters by maximizing the likelihood function based on observed data. In signal localization, ML leverages DFSs to precisely determine the position of the signal source. While highly accurate, it can be computationally expensive, especially in scenarios involving a large number of parameters or complex models [49].

The QN method is an iterative optimization approach that approximates Newton’s method without requiring the explicit computation of the Hessian matrix. By updating an approximation of the inverse Hessian matrix at each iteration, it achieves faster convergence compared to gradient-based methods. The QN is extensively used in solving convex optimization problems, offering a balance between computational efficiency and accuracy [50].

The CDFs of the location error for the four abovementioned DPD techniques presented in Figure 4 [15] are the basis of comparative analysis with the proposed approach. To extract the numerical data from this figure, the WebPlotDigitizer software 5.0 was applied [51].

**Figure 17:**
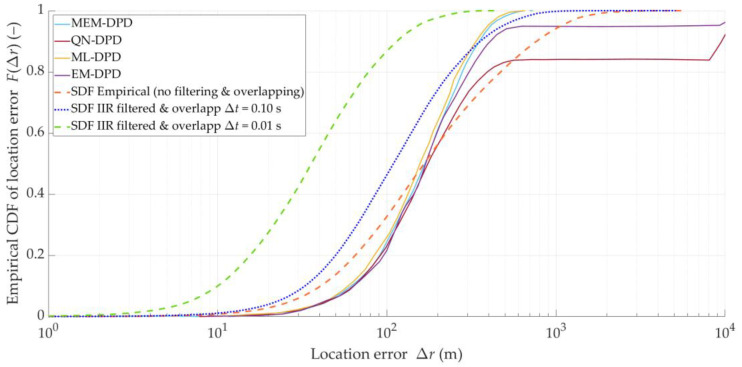
Empirical CDFs of location error for four analyzed DPD techniques and three SDF variants.

We want to assess the effectiveness of the SDF method in three variants, i.e., the SDF method based only on empirical data (i.e., no filtering and overlapping), the SDF method with DFS filtration and overlapping at the DFS vector level, and the SDF method using DFS filtering and two-level overlapping. We use the summary from Section 5.2, Section 5.3, Section 5.4 and Section 5.5 as the basis for this assessment. For the variant with the DFS filtering, we use the 16th-order Butterworth IIR filter with the cutoff frequency equal to $\omega_{C}=0.05F_{s}$. For the variant with overlapping at the DFS vector level, we adopt the following parameters: $t_{A}=\Delta t=0.1\;s$ (i.e., $O_{t}=100\%$), $T_{A}=20\;s$, and ΔT=1 s (i.e., $O_{T}=5\%$). For the variant with two-level overlapping, we use $t_{A}=0.1\;s$ and Δt=0.01 s (i.e., $O_{t}=10\%$), and $T_{A}=20\;s$ and ΔT=1 s (i.e., $O_{T}=5\%$). The empirical CDFs of the location error for the four analyzed DPD techniques (i.e., based on [15]) and three SDF variants are shown in Figure 17. Additionally, Table 6 provides the MIN, MED, AVG, and STD of Δr for the three SDF variants.

Based on Figure 17, we can see that the MEDs of Δr for the DPD methods are equal to about 160 m. Using this measure, it can be concluded that each SDF variant is more efficient than the analyzed DPD techniques. In the case of the SDF based only on empirical data (i.e., no filtering and overlapping), the shape of the CDF indicates that there are large location errors exceeding those of the two DPD methods (i.e., the MEM-DPD and ML-DPD). The maximum errors in the SDF variant with only filtering are similar to the MEM-DPD and ML-DPD. Meanwhile, the SDF with filtering and overlapping data is characterized by minor localization errors.

## 6. Conclusions

This paper presents the application of two data processing techniques, i.e., filtration and overlapping, which aim to enhance the accuracy of SDF localization. The presented analysis is based on simulation studies.

The filtering process was applied at the DFS vector level to reduce instantaneous fluctuations in the estimated DFS values, thereby improving localization accuracy. Three types of filters were analyzed, i.e., two FIR filters with the Hamming and Gaussian windows, as well as the IIR Butterworth filter. The results demonstrated that all three filter types provided comparable improvements in DFS estimation accuracy and localization performance. While the choice of filter type had a minimal impact on the final results, applying low-pass filtering significantly reduced localization errors compared to empirical DFS data, confirming its effectiveness in enhancing the precision of the SDF method.

In this paper, we presented the idea of the two-level overlapping algorithm. The first level of processing concerns the IQ sample of the radio signal, while the second concerns the vector of the estimated or filtered DFSs. The analysis was based on the empirical CDFs of the localization error accumulated for five different Tx positions. Based on the obtained results, it can be concluded that the application of the overlapping algorithm at the IQ sample level allows for more than a twofold increase in the SDF accuracy. The use of overlapping at the DFS vector level contributes primarily to an increase in the refresh rate of the position of the localized object, which is of significant importance in real localization systems. Moreover, the comparison of the SDF method with the DPD techniques showed the high effectiveness of the developed method, especially using the filtration and overlapping proposed in this paper.

The authors’ future work will focus on two aspects. First, we plan to use the research results to set appropriate operating parameters for localization sensors on UAV equipment. This will allow for conducting empirical measurements in a real environment and conducting a practical assessment of SDF localization efficiency. Second, we plan to develop an adaptive method for selecting sensor operating parameters depending on the Rx flight parameters (e.g., flight speed, distance to Tx), and to automate changes in its flight direction.

## Figures and Tables

**Table 1:** Comparison of absolute DFS errors for empirical data and three analyzed filter types.

**DFS Vector**	$F_{C}/F_{s}$**,** $\omega_{C}/F_{s}$ (–)	$MED\left({\Delta f}_{D}\right)$ $\left(Hz\right)$	$AVG\left({\Delta f}_{D}\right)$ $\left(Hz\right)$	$STD\left({\Delta f}_{D}\right)$ $\left(Hz\right)$
Empirical	–	4.05	4.79	3.61
Filtered by Gaussian FIR	0.05	1.25	1.61	2.24
Filtered by Hamming FIR	0.05	1.21	1.57	2.29
Filtered by Butterworth IIR	0.05	0.89	1.07	0.87
Filtered by Gaussian FIR	0.10	1.32	1.68	2.19
Filtered by Hamming FIR	0.10	1.29	1.64	2.23
Filtered by Butterworth IIR	0.10	1.27	1.51	1.16
Filtered by Gaussian FIR	0.25	1.80	2.20	2.08
Filtered by Hamming FIR	0.25	1.81	2.20	2.09
Filtered by Butterworth IIR	0.25	1.98	2.36	1.79

**Table 2:** Comparison of location errors for empirical data and three analyzed filter types with selected cutoff frequencies.

**DFS Vector**	$MIN\left(\Delta r\right)$ $\left(m\right)$	$MED\left(\Delta r\right)$ $\left(m\right)$	$AVG\left(\Delta r\right)$ $\left(m\right)$	$STD\left(\Delta r\right)$ $\left(m\right)$
Empirical	0.07	17.45	31.51	213.62
Filtered by Gaussian FIR	0.04	15.37	21.23	19.50
Filtered by Hamming FIR	0.01	15.32	21.14	19.41
Filtered by Butterworth IIR	0.03	15.18	20.82	19.05

**Table 3:** Comparison of SDF localization accuracy for four selected Δt.

$O_{t}$ (%)	Δt $\left(s\right)$	$MIN\left(\Delta r\right)$ $\left(m\right)$	$MED\left(\Delta r\right)$ $\left(m\right)$	$AVG\left(\Delta r\right)$ $\left(m\right)$	$STD\left(\Delta r\right)$ $\left(m\right)$
10	0.01	0.07	6.52	10.26	11.01
20	0.02	0.05	9.29	15.90	19.96
50	0.05	0.04	14.68	24.78	30.46
100	0.10	0.33	20.68	31.97	33.90

**Table 4:** Comparison of SDF localization accuracy for four selected ΔT.

$O_{T}$ (%)	ΔT $\left(s\right)$	$MIN\left(\Delta r\right)$ $\left(m\right)$	$MED\left(\Delta r\right)$ $\left(m\right)$	$AVG\left(\Delta r\right)$ $\left(m\right)$	$STD\left(\Delta r\right)$ $\left(m\right)$
10	1	0.13	14.33	19.98	19.67
20	2	0.13	14.31	20.26	20.20
50	5	0.19	14.05	19.18	19.36
100	10	0.19	14.55	20.29	19.71

**Table 5:** Comparison of SDF localization accuracy for four selected $T_{A}$.

$O_{T}$ (%)	$T_{A}$ $\left(s\right)$	$MIN\left(\Delta r\right)$ $\left(m\right)$	$MED\left(\Delta r\right)$ $\left(m\right)$	$AVG\left(\Delta r\right)$ $\left(m\right)$	$STD\left(\Delta r\right)$ $\left(m\right)$
200	5	0.16	27.64	39.42	42.73
100	10	0.08	14.30	20.06	18.92
50	20	0.06	7.97	12.00	12.46
33	30	0.07	6.20	11.36	13.59

**Table 6:** Comparison of location errors for three SDF variants.

**SDF Variant**	$MIN\left(\Delta r\right)$ $\left(m\right)$	$MED\left(\Delta r\right)$ $\left(m\right)$	$AVG\left(\Delta r\right)$ $\left(m\right)$	$STD\left(\Delta r\right)$ $\left(m\right)$
SDF without filtering and overlapping	0.03	146.45	274.52	291.75
SDF with filtering and overlapping at DFS vector level	0.32	105.49	157.35	158.48
SDF with filtering and two-level overlapping	0.06	36.30	52.98	52.20

## Data Availability

The data presented in this study are available on request from the corresponding author. The data are not publicly available due to project restrictions.

## Abbreviations

The following abbreviations are used in this manuscript:

2D	two-dimensional
2G	second-generation
3D	three-dimensional
3G	third-generation
4G	fourth-generation
AOA	angle of arrival
ASLER	autonomous system of locating radio emitters
AVG	average (mean) value
CDF	cumulative distribution function
COMINT	communication intelligence
DFS	Doppler frequency shift
DPD	direct position determination
ECG	electrocardiogram
EEG	electroencephalogram
EM-DPD	expectation maximization direct position determination
ESOLA	epoch-synchronous overlap-add
FDOA	frequency difference of arrival
FFT	fast Fourier transform
FIR	finite impulse response
FOA	frequency of arrival
FPGA	field-programmable gate array
GNSS	global navigation satellite system
IIR	infinite impulse response
IQ	in-phase and quadrature components/samples
IT	information technology
LBS	location-based service
LCS	location service
LOS	line-of-sight
LTE	Long-Term Evolution
MED	median
MEM-DPD	modified expectation maximization direct position determination
ML-DPD	maximum likelihood direct position determination
MIN	minimum
OFDM	orthogonal frequency division multiplexing
OLA	overlap-add
OLOS	obscured line-of-sight
PAOLA	peak alignment overlap-add
PCA	point of closest approach
POA	phase of arrival
PSOLA	pitch synchronous overlap-add
QN-DPD	quasi-Newton direct position determination
RSS	received signal strength
RTT	round-trip time
Rx	receiver
SAOLA	synchronized and adaptive overlap-add
SDF	signal Doppler frequency
SDR	software-defined radio
SNR	signal-to-noise ratio
SOLA	synchronous overlap-add
STD	standard deviation
STFT	short-time Fourier transform
TC-OLA	time-compression overlap-add
TDOA	time difference of arrival
TOA	time of arrival
TRL	technology readiness level
Tx	transmitter
UAV	unmanned aerial vehicle
UAV-COMINT	acronym of project on “Command and control of a group of COMINT radio-electronic reconnaissance unmanned aerial vehicles based on modern IT technologies”
WOLA	weighted overlap-add
